# Genome-scale model-driven strain design for dicarboxylic acid production in *Yarrowia lipolytica*

**DOI:** 10.1186/s12918-018-0542-5

**Published:** 2018-03-19

**Authors:** Pranjul Mishra, Na-Rae Lee, Meiyappan Lakshmanan, Minsuk Kim, Byung-Gee Kim, Dong-Yup Lee

**Affiliations:** 1 NUS Synthetic Biology for Clinical and Technological Innovation (SynCTI), Life Sciences Institute, National University of Singapore, 28 Medical Drive, Singapore, 117456 Singapore; 2 Bioprocessing Technology Institute, Agency for Science, Technology and Research (A*STAR), 20 Biopolis Way, #06-01, Centros, Singapore, 138668 Singapore; 3 School of Chemical and Biological Engineering, Institute of Molecular Biology and Genetics, and Bioengineering Institute, Seoul National University, 1 Gwanak-ro, Gwanak-gu, Seoul, 151-742 Republic of Korea; 4 School of Chemical Engineering, Sungkyunkwan University, 2066 Seobu-ro, Jangan-gu, Suwon, Gyeonggi-do 16419 Republic of Korea

**Keywords:** *Yarrowia lipolytica*, Dicarboxylic acid, Genome-scale metabolic models, Strain design, Metabolic engineering

## Abstract

**Background:**

Recently, there have been several attempts to produce long-chain dicarboxylic acids (DCAs) in various microbial hosts. Of these, *Yarrowia lipolytica* has great potential due to its oleaginous characteristics and unique ability to utilize hydrophobic substrates. However, *Y. lipolytica* should be further engineered to make it more competitive: the current approaches are mostly intuitive and cumbersome, thus limiting its industrial application.

**Results:**

In this study, we proposed model-guided metabolic engineering strategies for enhanced production of DCAs in *Y. lipolytica*. At the outset, we reconstructed genome-scale metabolic model (GSMM) of *Y. lipolytica* (*i*YLI647) by substantially expanding the previous models. Subsequently, the model was validated using three sets of published culture experiment data. It was finally exploited to identify genetic engineering targets for overexpression, knockout, and cofactor modification by applying several *in silico* strain design methods, which potentially give rise to high yield production of the industrially relevant long-chain DCAs, e.g., dodecanedioic acid (DDDA). The resultant targets include (1) malate dehydrogenase and malic enzyme genes and (2) glutamate dehydrogenase gene, *in silico* overexpression of which generated additional NADPH required for fatty acid synthesis, leading to the increased DDDA fluxes by 48% and 22% higher, respectively, compared to wild-type. We further investigated the effect of supplying branched-chain amino acids on the acetyl-CoA turn-over rate which is key metabolite for fatty acid synthesis, suggesting their significance for production of DDDA in *Y. lipolytica*.

**Conclusion:**

*In silico* model-based strain design strategies allowed us to identify several metabolic engineering targets for overproducing DCAs in lipid accumulating yeast, *Y. lipolytica*. Thus, the current study can provide a methodological framework that is applicable to other oleaginous yeasts for value-added biochemical production.

**Electronic supplementary material:**

The online version of this article (10.1186/s12918-018-0542-5) contains supplementary material, which is available to authorized users.

## Background

Long-chain dicarboxylic acids (DCAs) are widely used in the manufacturing of polyamides and polyesters as the monomeric intermediates [1]. The most commonly employed chemical process to produce DCAs is the ring-opening oxidation of cyclic compounds. However, it requires expensive starting material as well as the environmentally hazardous procedures [2]. Alternatively, DCAs can be synthesized through bio-routes in various microbial hosts, such as *Candida* sp. [3, 4], *Yarrowia lipolytica* [5], *Pseudomonas aeruginosa* [6], *Cyptococcus neoformans* [7], and *Escherichia coli* [8]. Of these, recently, *Y. lipolytica* has attracted great attention as a cell factory to manufacture DCAs [9] since this oleaginous yeast is capable of accumulating large amounts of lipids and possess a unique ω-oxidation pathway to catalyze the hydrophobic substrates such as n-alkane and fatty acids [10–13]. In ω-oxidation pathway, after the oxidation of ω-terminal of the fatty acid, fatty acid aldehyde is produced from ω-hydroxy fatty acid by fatty alcohol oxidase, followed by its oxidization to DCAs by NAD-dependent fatty aldehyde dehydrogenase [14].

Indeed, *Y. lipolytica* as oleaginous yeast has huge potential to become a model organism to produce fatty-acid derived products including DCAs. In order to make it industrially competitive, its productivity should be further enhanced, which can be achieved by modification of relevant target genes. However, metabolic engineering of *Y. lipolytica* has been mainly focused on over-producing lipids, e.g., triacylglycerols (TAGs) with only a handful of studies for the increased DCAs production [15]. In addition, most of genetic engineering targets have been identified in an intuitive or ad hoc manner, which limited our design scope for strain improvement. Therefore, it is now imperative to adopt more rational systems approaches [16]. In this regard, various strain design strategies guided by *in silico* genome-scale metabolic model (GSMM) have been developed and successfully applied to several industrial hosts including *E. coli* [17, 18] and *S. cerevisiae* [19, 20]. Similarly, in this work, we exploited such *in silico* methods using GSMM of *Y. lipolytica* which was newly reconstructed by substantially expanding the previous models, thus allowing us to identify various genetic engineering targets for overexpression, knockout, and cofactor modification towards DCA overproduction.

## Results and discussion

### Genome-scale metabolic reconstruction of *Y. lipolytica*

**Fig. 1:**
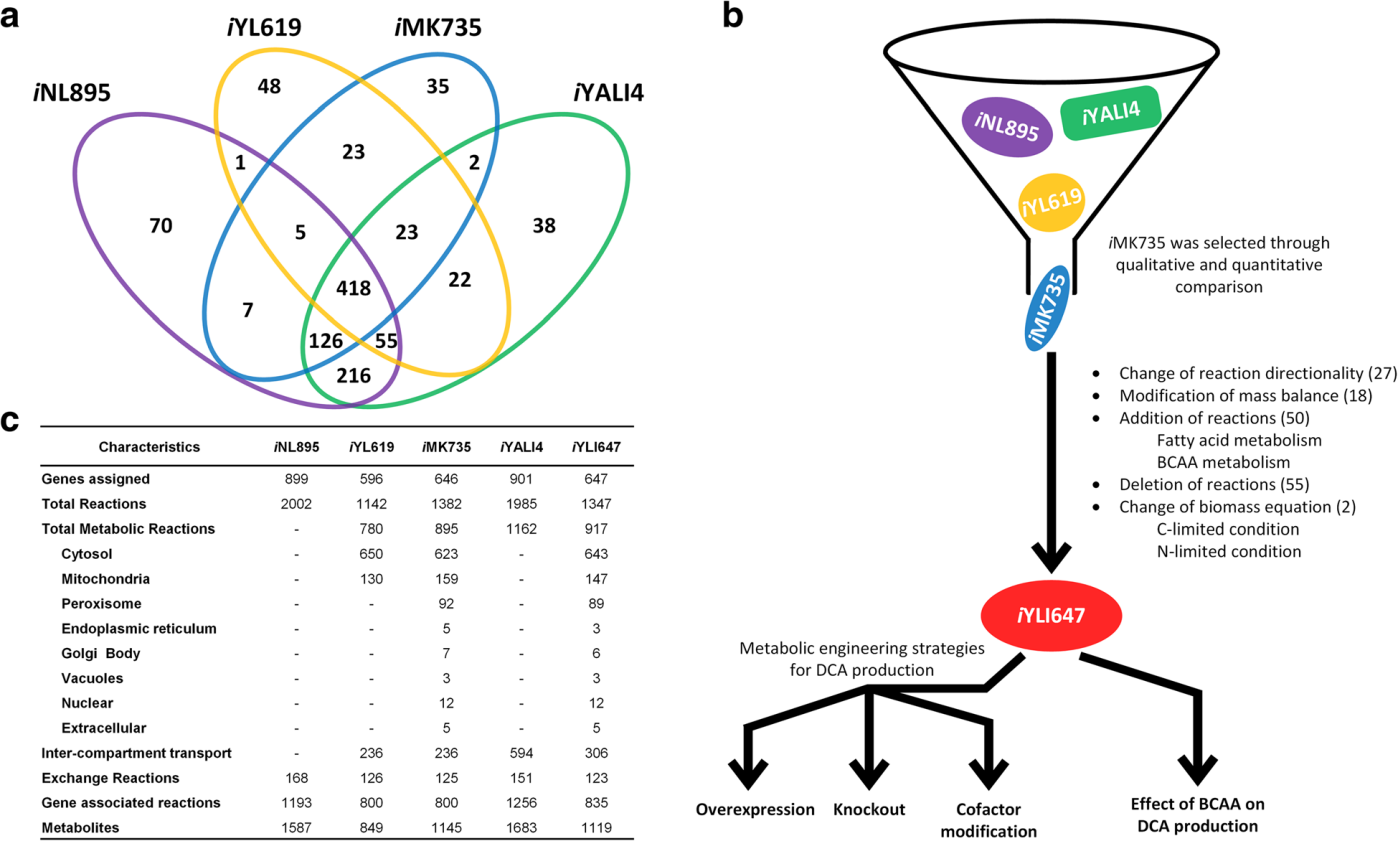
Reconstruction process and characteristic of *in silico* models **a** Comparison of previous *Y. lipolytica* models **b** Schematic diagram highlighting the overall reconstruction process of *i*YLI647, followed by application for strain designing **c** General features of *i*YLI647 in comparison with previous four models

We have reconstructed a genome-scale metabolic model of *Y. lipolytica* for designing DCA overproducing strains. Initially, four existing *Y. lipolytica* GSMMs (*i*NL895 [21], *i*YL619 [22], *i*MK735 [23] and *i*YALI4 [24]) were compared on the basis of their gene annotations (see Methods and Fig. 1a). While *i*NL895 was developed from the phylogenetically distant yeast *S. cerevisiae* model, *i*YL619 was reconstructed based on biochemical information databases such as KEGG and BRENDA. *i*MK735 was completely derived from *S. cerevisiae* model *i*ND750 [25] by adding a few reactions related to alkane uptake and lipid metabolism whereas *i*YALI4 stands a bit different from other three models since it was built automatically using RAVEN toolbox from yeast consensus model as template. After qualitative and quantitative assessment, *i*MK735 was selected as suitable scaffold model since it has wider coverage of cellular physiology of *Y. lipolytica* with better quality in terms of representation and prediction quality.

With the aim of building an objective-oriented model for simulating DCAs production, we first added 9 biosynthetic reactions which are sequentially catalyzed by hydroxylase, oxidase and dehydrogenase enzymes within the ω-oxidation pathway, thus capturing the oxidation of fatty acids to DCAs. The 27 DCAs degradation reactions were also included to represent β-oxidation pathway together with 6 dicarboxylic acids transport and an exchange reaction pertaining to DDDA secretion. Based on the scaffold model, *Y. lipolytica* appeared to not degrade leucine and other BCAA into acetyl-CoA, but there are reports implicating the biosynthetic capacity of leucine as an effector of lipogenic capacity in oleaginous organisms [26]; a putative acetyl-CoA producing leucine degradation pathway was recently identified [27]. Therefore, we have added 4 relevant enzymatic reactions for leucine degradation in the model. In total, we added 50 new reactions to the scaffold model mainly related to ω-oxidation and BCAA degradation.

During the manual curation, we removed 55 reactions which are responsible for dead-ends or futile cycles without literature evidence. For example, lactaldehyde dehydrogenase was deleted since there is not enough experimental evidence to support the existence of such reaction in *Y. lipolytica*. Additionally, we corrected the elemental balance and directionality of 45 reactions. For example, the directionality of THRA representing the threonine aldolase mediated breakdown of threonine to glycine and acetaldehyde, was changed to opposite direction favoring acetaldehyde formation based on the direction reported in *S. cerevisiae* [28]. All the changes made from the scaffold are provided in Additional file 1 and summarized in Fig. 1b. Finally, the resulting *in silico* *Y. lipolytica* model (*i*YLI647) consists of 1347 reactions and 1152 metabolites encoded by 647 genes (Fig. 1c). The *i*YLI647 is available as Systems Biology Markup Language (SBML) file (Additional file 2).

The model predictability of cell growth relies highly on the accuracy of biomass equation. However, we found that the biggest shortcoming of the previous models is the inaccurate biomass composition since they mainly used the information of *S. cerevisiae* biomass. Hence, the whole biomass compositions of *Y. lipolytica* were derived based on various *Y. lipolytica* experimental data published under carbon limited and nitrogen limited conditions which can significantly alter the amino acid and lipid composition. The calculated compositions were then assimilated into *i*YLI647 as two separate biomass synthesis equations pertaining to C-limited and N-limited conditions (Additional file 3). Growth and non-growth associated ATP maintenance (GAM and NGAM) requirements for cellular processes were also derived using relevant literature data [29]. The NGAM of *Y. lipolytica* was estimated to be 5.03 mmol ATP/gDCW, and GAM was 23.09 mmol ATP/gDCW.

### Comparative validation of *i*YLI647 with Other *Y. lipolytica* GSMMs

**Fig. 2:**
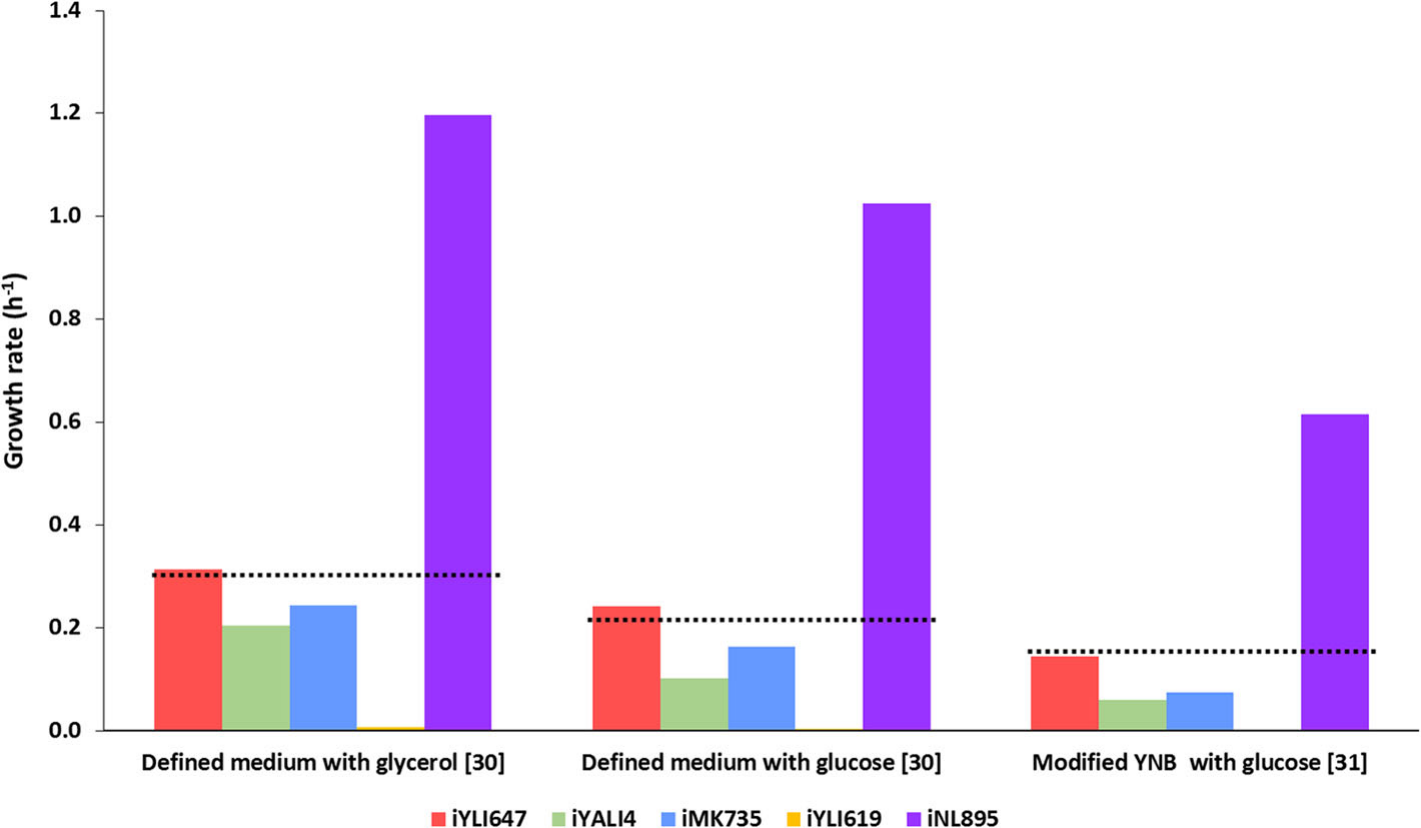
Comparative validation of *i*YLI647 with all 4 *Y. lipolytica* models available under 3 different datasets. The dotted lines represent experimental value

The *i*YLI647 was evaluated for its predictions correlated with experimental phenotypes. The growth predictions under different culture conditions of *i*YLI647 were compared with those for other *Y. lipolytica* GSMMs. To do so, the *in silico* biomass yields of all the models on glucose and glycerol minimal medium under steady-state conditions were predicted using FBA. For each set of culture data taken from independent studies, the carbon source uptake rates were constrained accordingly, while maximizing biomass. Additionally, the CO_2_ evolution rate (CER) was also constrained according to the experimental data wherever provided. For the biomass maximization in *i*MK735, biomass equation corresponding to 5% lipid was used. The maximum specific growth rate was determined for all models simulated under same constraints and the results were compared (Fig. 2).

Datasets 1 and 2 were taken from Workman et al., [30] in which *Y. lipolytica* was grown in batch culture on glycerol and glucose as a sole carbon source, respectively. During the batch culture with glucose, only biomass and CO_2_ were produced as no metabolite production was observed. In the case of glycerol, apart from biomass and CO_2_, small amount of polyols in the form of mannitol and arabitol, were also produced. Since, the individual breakdown of polyols was not mentioned in the literature and amounts were small enough to drastically affect the result, we didn’t take it into consideration for simulation. Dataset 3 was taken from Dulermo et al., [31] which endeavored to analyze the *Y. lipolytica* mutants for fatty acid production. The carbon source uptake and CER were constrained as per the values reported in the literature and biomass was maximized.

It can be seen from comparative validation that *i*YLI647 can predict the evaluated macroscopic growth parameters more accurately, i.e. smaller deviations from experimental data shown in black dotted lines, as compared to other models. We believe the growth predictions are directly influenced by the accuracy of the biomass composition, and especially, in case of *Y. lipolytica* the biomass composition can drastically change depending on the culture condition. Another important factor that affects the predictions is the GAM and NGAM values used in the model. Although, in comparative prediction done in the current study, we have used same NGAM in all the models but GAM value was unchanged, and some models reported very high GAM value, resulting in discrepancies in the model prediction.

### Metabolic engineering strategies for DCAs production

**Fig. 3:**
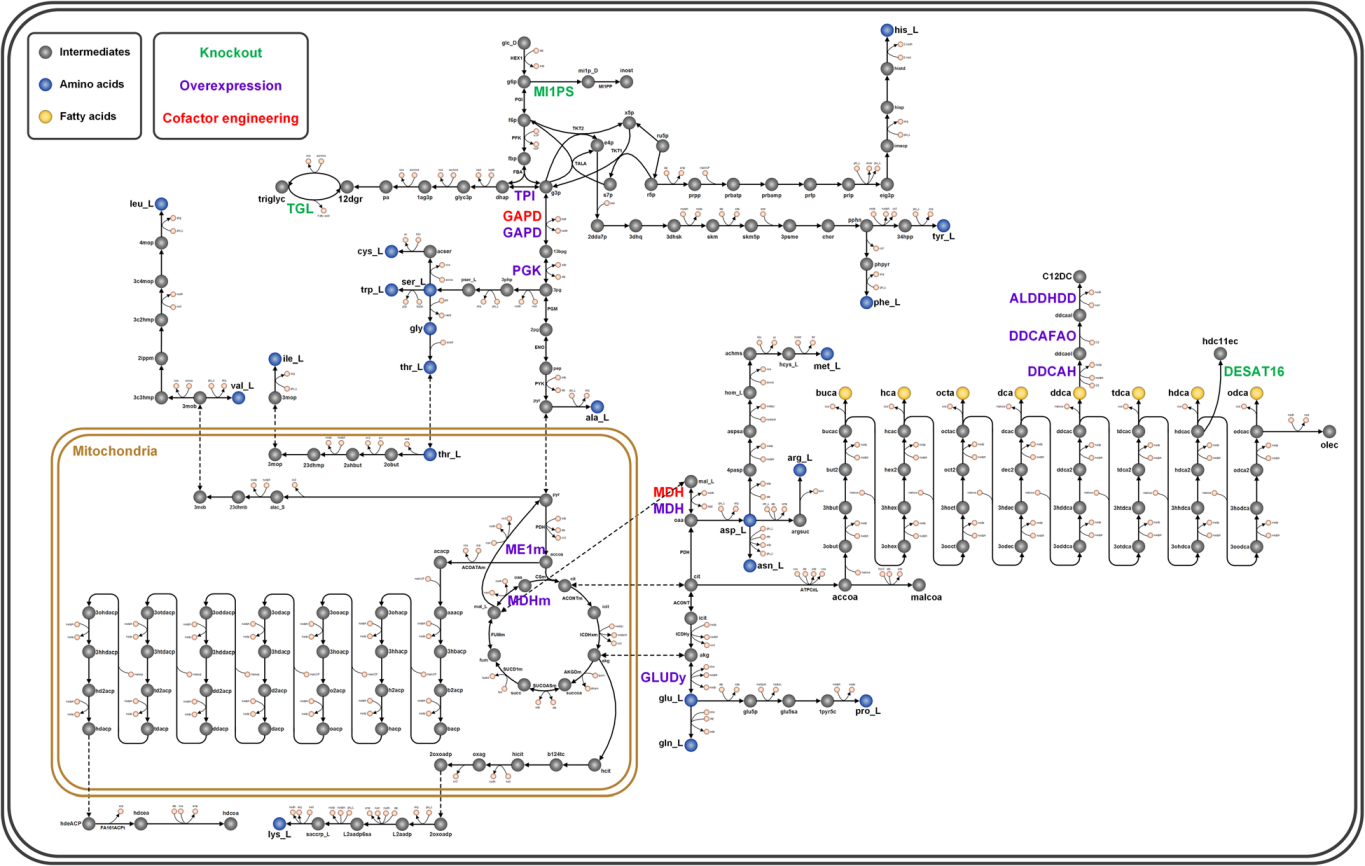
The central metabolic network of *Y. lipolytica* depicting metabolic engineering targets to produce DDDA

In the oleaginous organism, de novo accumulation of lipids starts with the formation of anabolic acetyl-CoA via glycolysis. The fatty acids formed by these acetyl-CoA get esterified to form TAGs [32]. A high number of carbon sources excluding cellulose and methanol, have been considered as substrates for the de novo DCAs biosynthesis in oleaginous microorganisms. Among the various carbon sources available, we used glucose to design the metabolic engineering strategies for de novo overproduction of DDDA, a representative of long-chain DCAs. Herein, we applied three model-guided design strategies to overproduce DDDA using four tools. First, we employed genetic design by local search (GDLS) [33] to find the growth-coupled solution to overproduce DCAs by knocking out a set of reactions. Then, we used flux activity analysis [34] to identify the bottlenecks in the metabolic network which can be considered as potential overexpression targets. In addition, we implemented transcriptomic-based strain optimization tool (tSOT) [35] which first generates activated reactions as a reference state to identify the deactivated reactions, addition of the each deactivated reactions can then lead to the increase in the product yield. Finally, to supplement the metabolic engineering targets with cofactor availability, we performed cofactor modification analysis (CMA) [36] to find the cofactor specificity engineering targets that can increase the pool of cofactors required for catalyzing the reactions. Figure 3 illustrates the central metabolic network of *Y. lipolytica* and identified genetic engineering targets for enhancing DCAs production.

#### Overexpression targets

**Fig. 4:**
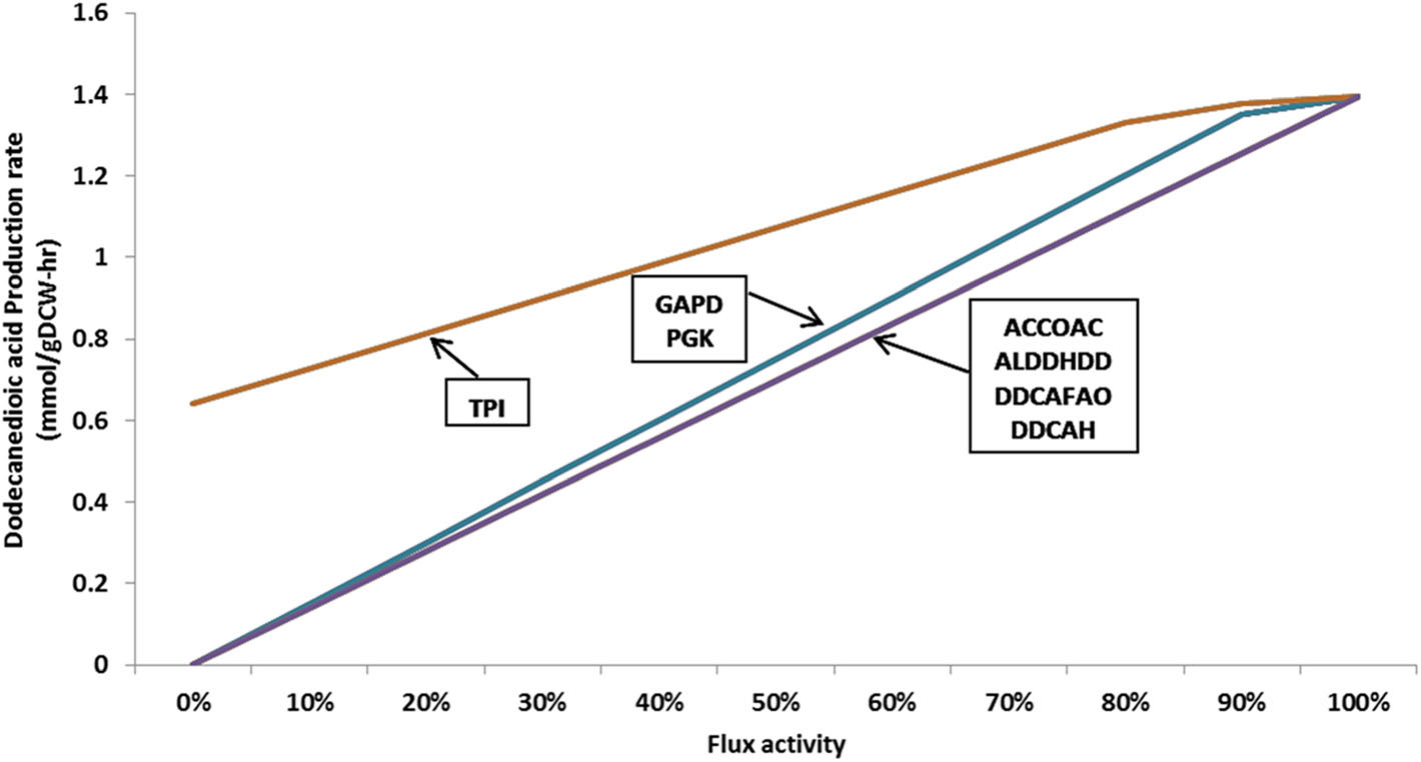
Simulation result by flux activity analysis. Overexpression genes and production rate changes depend on the alteration of flux activities of respective genes. GAPD (Glyceraldehyde-3-phosphate dehydrogenase), PGK (Phosphoglycerate kinase), TPI (Triose phosphate kinase), ACCOAC (Acetyl-CoA carboxylase), ALDDHDD (Aldehyde dehydrogenase), DDCAFAO (Fatty acid oxidase), DDCAH (Fatty acid hydroxylase)

Expression level of genes and the activity of their enzyme products are highly optimized to meet the performance demand of a biological system [37]. However, these enzyme expressions alter to adapt against the changing biological conditions. This range of fluctuations in gene expression level can be exploited to design an overexpression system [38]. Based on this principle, we performed flux activity analysis under glucose minimal medium by fixing an optimal biomass and systematically increasing flux activity of each reaction from 0% to 100% and maximizing DDDA. Simulation results show that when *Y. lipolytica* growing in 10 mmol/gDCW-hr of glucose, the increase in flux activity of some reactions has proportional effect on the maximum achievable yield of DDDA (Fig. 4). These directionally coupled reactions can be genetically overexpressed to complement the ω-oxidation pathway enzymes to produce DDDA at its theoretical maximum. Some of the bottleneck reactions, e.g., DDCAH, a cytochrome P450 hydroxylase (CYP52) and DDCAFAO, a fatty alcohol oxidase identified by *in silico* analysis have been verified as overexpression targets for DDDA production [39]. The other hypothesized reactions include acetyl-CoA carboxylase (ACCOAC), overexpression of which may increase the malonyl-CoA pool. It can be further utilized by FAS complex to generate more fatty acids, which can then be channelized to ω-oxidation pathway.

Considering that fatty acid biosynthetic pathway is very tightly regulated, transcriptomic data can provide some useful insight into the ON/OFF state of the reactions in a particular condition. To identify the overexpression targets for increasing the DCAs production, we implemented tSOT by resorting to time-course transcriptomic profile of the *Y. lipolytica*, during a controlled fed-batch using glucose as the sole carbon source after 27 h time-point which corresponds to early stationary phase [40]. A nitrogen limitation was applied during the fed-batch to initiate de novo lipid synthesis which can represent the precondition for high DCAs production. The basic principle of tSOT is to ascertain the gene overexpression targets by restoring the reactions which are removed from GSMM by data-integration algorithms while developing a context-specific model. As a result, tSOT identified MDH, both cytosolic and mitochondrial, as an overexpression target. In addition, it also found mitochondrial NAD-dependent malic enzyme (ME1m) and glutamate dehydrogenase (GLUDy) to be the overexpression targets (Table 1). Interestingly, owing to the fact that ω-oxidation is an oxidative process with the high demand of redox cofactors, all the identified reactions are involved in cofactor regeneration. ME is hypothesized to be the supplier of NADPH during lipid biosynthesis in most oleaginous yeasts through the intracellular substrate cycles involving MDH, pyruvate carboxylase (PC) and ME, also called “transhydrogenase cycle” [41]. Although *Y. lipolytica* lacks a cytosolic copy of ME required to compensate for NADPH demand, it could be interesting to investigate the compound effect of overexpressing MDH and ME because apart from NADPH, ME in conjunction with mitochondrial pyruvate dehydrogenase (PDH) also provide mitochondrial acetyl-CoA. The most interesting finding from tSOT is GLUDy, which apart from regeneration of NADPH; also plays key role in maintaining the balance of carbon and nitrogen. There is considerable evidence of the presence of GLUDy shunt in plants, which returns the carbon in amino acids biosynthesis back into reactions of carbon metabolism and TCA cycle [42] which is the case in nitrogen starving condition [24], making it particularly an interesting target to explore as it links amino acid biosynthesis to fatty acid metabolism.

**Table 1:** Overexpression targets simulated by tSOT to increase DCAs production

Targets	Reaction Name	Reaction Definition	Yield Improvement (%)
GLUDy	Glutamate Dehydrogenase (NADPH-forming)	glu_L[c] + h2o[c] + nadp[c] < => akg[c] + h[c] + nadph[c] + nh4[c]	22.2
MDH	Malate Dehydrogenase (cytosol)	mal_L[c] + nad[c] < => h[c] + nadh[c] + oaa[c]	47.8
MDHm	Malate Dehydrogenase (mitochondrial)	mal_L[m] + nad[m] < => h[m] + nadh[m] + oaa[m]	47.8
ME1m	Malic enzyme (NAD-dependent)	mal_L[m] + nad[m] - > co2[m] + nadh[m] + pyr[m]	47.8

#### Downregulation/knockout targets

GDLS algorithm was used to search for growth-coupled solutions for DCA production, identifying up to 5 reaction deletion candidates. Basically, GDLS propose the pathway design which couples the product formation with the cell growth, making its production necessary to reach optimal growth. The strain design strategy deciphered by GDLS combined the simultaneous knockout of DESAT16 (Stearoyl-CoA desaturase) and MI1PS (Myo-inositol-1-phosphate synthase). DESAT16 is the enzyme that catalyzes the conversion of saturated fatty acid to monounsaturated fatty acids. The overexpression of this enzyme is shown to increase lipid accumulation in *Y. lipolytica* which apparently takes the flux away from DCAs production which requires free fatty acid [43], deleting which can result in increased pool of free fatty acids. Since DCAs is a non-growth coupled product, deletion of DESAT16 and MI1PS as suggested by GDLS may not give rise to improved product yield. However, in the simple network perspective, DESAT16 and MI1PS reactions branch the carbon flux away from DCAs formation which makes them interesting knockout targets to test experimentally. It is worth noticing here that MI1PS catalyzes the first reaction in the inositol pathway which produces membrane forming metabolites, so knocking out MI1PS could show deleterious effects on cell growth. Nonetheless, since DCAs is non-growth associated product, an inducible knockout strain could be used wherein MI1PS is suppressed when the culture reaches stationary phase.

Furthermore, formed DCAs can get degraded via β-oxidation pathway. Therefore blocking it by deletion of acyl-CoA oxidase encoded by POX1–6 genes can further enhance the titer of DCAs. *Y. lipolytica* which lacks acyl-CoA oxidases can more efficiently convert n-alkanes and fatty acids or their derivatives to their corresponding DCAs [5].

#### Cofactor specificity engineering targets

Similar to other biosynthetic pathways, the DCAs biosynthesis via ω-oxidation pathway in *Y. lipolytica* involves several unique reactions and is commonly controlled by the supply of precursors and cofactors. Earlier part of this study has focused on overexpression and downregulation of some key enzymes regulating the ω-oxidation pathway. However, cofactors are very important to achieve improvement in productivity. NADPH, as a reducing equivalent, usually plays an important role in coupling catabolism with anabolism and energy generation during metabolism. Several metabolic engineering approaches have been implemented to manipulate the cofactors level to increase the product yield in other microorganisms [44, 45] Increasing the α-santalene production by modifying the ammonium assimilation from being NADPH to NADH dependent by the deletion of GDH1 and the overexpression of GDH2; overexpression of *Streptococcus* mutants gapN gene which encodes a GAPDH to increase the L-lysine production [46]; engineering NADPH regeneration for improving pentose fermentation by overexpressing the GDP1, a NADP-dependent GAPDH from *Kluyveromyces lactis* [47]; overexpression of transhydrogenase and NAD kinase to improve isobutanol production [48], are a few examples showing the emergence of NADPH level engineering as potent and feasible strategy to increase the production in microbial hosts.

Fatty acid biosynthesis and ω-oxidation pathway are NADPH demanding oxidative pathway in *Y. lipolytica*. This excessive cofactor demand is mainly satisfied through the pentose phosphate pathway (PPP) reactions. But, the increased usage of the PPP through overexpression is suboptimal as one mole of carbon is lost as CO_2_ for every two moles of NADPH produced [49]. To circumvent this, we performed CMA in order to identify the targets for cofactor specificity engineering to improve the NADPH pool which can enhance DDDA yield. From the CMA results, it was observed that the DDDA yield can be improved by increasing NADPH regeneration through modification of cofactor specificity from NAD to NADP. Among the targets found, changing the cofactor specificity of GAPD and MDH from NAD to NADP was found to give the best improvement in DDDA yield (Table 2).

**Table 2:** Cofactor specificity engineering targets by CMA to increase DCAs production

Reaction Name	Reaction Definition	Yield of DDDA (mmol/gDCW-hr)	
		NAD	NADP
MDH	mal_L[c] + nad[c] < => h[c] + nadh[c] + oaa[c]	2.601	2.781
GAPD	g3p[c] + nad[c] + pi[c] < => 13dpg[c] + h[c] + nadh[c]	2.601	2.778

To achieve a high-level production of DCA in yeast, we propose to enhance the supply of NADPH to the ω-oxidation pathway. Hence, a multi-step metabolic engineering strategy can be devised to simultaneously modify the glycolysis step, optimize the NADPH supply and to enhance the activity of some NADPH producing enzymes.

### Effect of branched-chain amino acids supplementation on DCA production

**Fig. 5:**
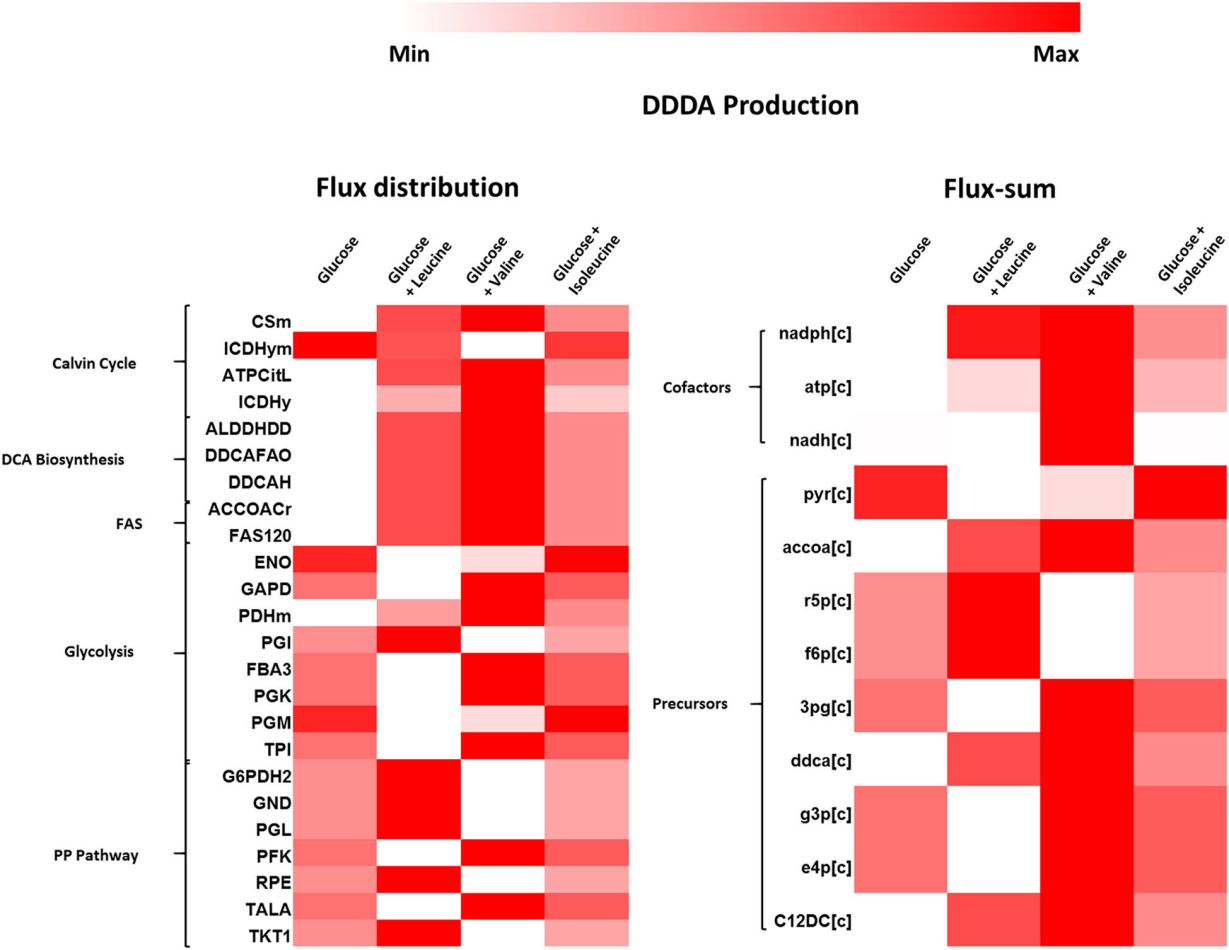
Flux distribution and flux-sum. Heat map showing flux distribution and flux-sum of important reactions and metabolites, respectively, during DDDA production

In most recombinant DCAs production studies, the hydrophobic substrates such as alkane and fatty acid methyl ester (FAME) were used for the biotransformation [5]. As such, glucose is used as the carbon source for the growth before inducing the ω-oxidation pathway using alkane. However, it has been shown in *Y. lipolytica* that lipid accumulation can occur de novo without exogenous supply of hydrophobic substrate, laying the grounds for a possibility to be used for de novo DCAs production as well, from primary carbon sources, i.e., glucose or glycerol [50]. In addition to this, the ability to efficiently produce acetyl-CoA by metabolizing other carbon sources in the medium will drive the important precursor towards DCAs production. Presently, there are evidences implicating a strong correlation between lipid accumulation and leucine metabolism in *S. cerevisiae* [51] and *Y. lipolytica* [52]. Taking the clue from lipid accumulation studies on *Y. lipolytica*, we sought to explore the effect of BCAA supplementation on acetyl-CoA pool, which can be produced via degradation pathway of BCAA. In order to theoretically analyze the flux distribution and flux-sum changes that occur when BCAA is supplemented, we maximized the DDDA production using glucose as the primary carbon source supplemented with 10 C-mmol/gDCW-hr of leucine, isoleucine, or valine. To elucidate the difference in pathway utilization and metabolite turn-over in different amino acids during DDDA production, we prepared the heat map of flux distribution and flux-sum as shown in Fig. 5. It can be seen that acetyl-CoA being critical determinant of DCAs synthesis was produced at the highest level on valine supplementation followed by leucine which correlated with DDDA turn over trend. Apart from the role of BCAAs in acetyl-CoA production, supplementing amino acids decreases the primary carbon demand in amino acid biosynthesis for biomass formation. This extra carbon can then be diverted to DCAs biosynthesis. This observation is in close resemblance with nitrogen starvation condition for lipid accumulation because in nitrogen limiting condition, biosynthesis of amino acid seizes and carbon present then can be utilized in other biosynthetic pathways. From our simulation, we have identified that glucose supplemented with valine or leucine can increase the growth as well as de novo accumulation of DCAs in *Y. lipolytica.* This is the first report implicating a prominent role of valine degradation as an alternate route of acetyl-CoA biosynthesis in *Y. lipolytica*, experimental validation of which can give insight to intricate correlation between fatty acid biosynthesis and amino acid degradation.

## Conclusion

The GSMM of *Y. lipolytica*, *i*YLI647, was reconstructed using *i*MK735 as a scaffold and the relevant information from other available *Y. lipolytica* models. *i*YLI647 consists of 1347 reactions and 647 genes. The biomass equations were carefully formulated with various experimental information of *Y. lipolytica*, which is perhaps the reason for accurate model prediction. The potential of de novo DCAs production in *Y. lipolytica* combined with a model–driven strain design for metabolic engineering and media optimization strategies were then evaluated using the reconstructed *i*YLI647. The flux towards DDDA production was increased following the overexpression and deletion of few reactions and the model-based strain design gives us a good starting point to explore the metabolic capabilities of *Y. lipolytica* to produce fatty acid derived products. Moreover, the workflow and procedure presented in this analysis can be utilized as a platform to perform similar analyses with different organisms.

## Methods

### Metabolic network reconstruction

We chose all four separately developed publicly available metabolic network reconstructions, *i*NL895, *i*YL619, *i*MK735, and *i*Yali4, to compare and select the best suited as scaffold model. Following our qualitative and quantitative comparison, we chose *i*MK735 as a scaffold model and proceeded to manual curation to expand the model coverage and characteristics. To do this, using literature data, we verified the presence of reactions and relevance in *Y. lipolytica* metabolism. Then, we added ω-oxidation pathway to convert fatty acids to DCAs, and also the subsequent degrading β-oxidation pathway. Following the literature evidence and its established importance in fatty acid metabolism, we also included BCAA degradation pathways. Furthermore, we checked the mass balance of reactions and made appropriate changes to make stoichiometrically balanced reactions. In addition, we derived the biomass equation in carbon and nitrogen limited conditions using updated and relevant literature sources. Using refined biomass equation, we simulated the model to identify the loops, and missing link reactions using GapFinder [53]. Loops were removed by changing the directionality of coupled reaction, or removing the non-metabolic lumped reactions, whereas, gaps were filled with reactions from orthologs or introducing transport/sink reaction.

### Constraints-based flux analysis

The cellular metabolism of *Y. lipolytica* was simulated under varying environmental conditions using constraints-based flux analysis. The biomass reaction was maximized to simulate the growth under various culture conditions as described elsewhere [54–56]. The maximization of biomass is subjected to stoichiometric and capacity constraints, which can mathematically be formulated as:

1 $$ \max \kern1em {Z}_1=\sum \limits_j{c}_j{v}_j $$

$$ \mathrm{s}.\mathrm{t}.\kern1em \sum \limits_j{S}_{ij}{v}_j=0\kern1em \forall \kern0.5em \mathrm{metabolite}\kern0.5em i $$

$$ {v}_j^{\mathrm{min}}\le {v}_j\le {v}_j^{\mathrm{max}}\kern1em \forall \kern0.5em \mathrm{reaction}\kern0.5em j $$

where *S*_*ij*_ refers to the stoichiometric coefficient of metabolite *i* involved in reaction *j*, *v*_*j*_ denotes to the flux or specific rate of metabolic reaction *j*, ${v}_j^{\mathrm{min}}$ and ${v}_j^{\mathrm{max}}$ represent the lower and upper limits on the flux of reaction *j*, respectively; and *Z*_*1*_ corresponds to the cellular objective as a linear function of all the metabolic reactions where the relative weights are determined by the coefficient *c*_*j*_. In this study, the constraints-based flux analysis problems were solved using COBRA toolbox [57].

### *In silico* model-based strain design

Four *in silico* strain design approaches have been employed to identify metabolic engineering targets for overproducing DCA from glucose.

#### Genetic design by local search (GDLS)

GDLS strain design algorithm [33] was implemented in COBRA toolbox in Matlab, searching up to maximum of 5 knockout reactions with the outer objective of maximizing DDDA exchange flux.

#### Flux activity analysis

To identify the upregulation and downregulation gene/reaction targets which lead to the enhanced production of desired compound, we first needed to quantify the flux activity of all reactions in the wild-type strain. Flux activity, *f*_*j*,_ is defined as the absolute value of reaction flux, *v*_*j*_ [34]. This can be determined by first solving the constraints-based flux analysis problem (Eq. 1) with biomass maximization as objective, and then obtaining the absolute values of individual reaction fluxes. Next, we solved the below-mentioned mixed-integer linear programming (MILP) problem to identify the maximum and minimum flux activities to determine the feasible ranges of individual reactions such that they can be upregulated and downregulated within this limit:

2 $$ \max /\min \kern1em {f}_j $$

Subject to:

$$ \sum \limits_j{S}_{ij}{v}_j=0 $$

$$ {\alpha}_j\le {v}_j\le {\beta}_j $$

$$ v{}_j={f}_j^{+}-{f}_j^{-} $$

$$ {f}_j^{+}\ge 0\kern1em ;\kern1em {f}_j^{-}\ge 0 $$

$$ {f}_j^{+}\le {I}_j^{+}\cdot M\kern1em ;\kern1em {f}_j^{-}\le {I}_j^{-}\cdot M $$

$$ {I}_j^{+}\in \left\{0,1\right\}\kern1em ;\kern1em {I}_j^{-}\in \left\{0,1\right\} $$

$$ {I}_j^{+}+{I}_j^{-}=1 $$

Where, *α*_*j*_ and *β*_*j*_ are upper and lower bounds of fluxes, respectively. The *f*_*j*_^+^ and *f*_*j*_^−^ are the positive two variables into which the flux, *v*_*j*_, is decomposed. It is observed that ${f}_j^{+}+{f}_j^{-}=\left|{f}_j^{+}-{f}_j^{-}\right|$, if and only if either *f*_*j*_^+^ or *f*_*j*_^−^ is equal to zero. This condition was introduced by new binary variables *I*_*j*_^+^ and *I*_*j*_^−^ which are when multiplied with a large integer which should be larger than the flux of experimentally measured value, M, will be always lesser than *f*_*j*_^+^ and *f*_*j*_^−^, respectively. Additionally, the constraint ${I}_j^{+}+{I}_j^{-}=1$ is also introduced to ensure that either *f*_*j*_^+^ or *f*_*j*_^−^ are equal to zero.

Once the reference flux activities are established, i.e. wild-type, maximum and minimum values, we then solve the below mentioned MILP problem to analyze the effects of perturbing a particular flux activity on cellular growth.

3 $$ \max \kern1em {v}_{biomass} $$

Subject to:

$$ \sum \limits_j{S}_{ij}{v}_j=0 $$

$$ {\alpha}_j\le {v}_j\le {\beta}_j $$

$$ v{}_j={f}_j^{+}-{f}_j^{-} $$

$$ {f}_j^{+}\ge 0\kern1em ;\kern1em {f}_j^{-}\ge 0 $$

$$ {f}_j^{+}\le {I}_j^{+}\cdot M\kern1em ;\kern1em {f}_j^{-}\le {I}_j^{-}\cdot M $$

$$ {I}_j^{+}\in \left\{0,1\right\}\kern1em ;\kern1em {I}_j^{-}\in \left\{0,1\right\} $$

$$ {I}_j^{+}+{I}_j^{-}=1 $$

$$ \left(\mathrm{C}1\right):\kern0.5em {f}_j^{+}+{f}_j^{-}\le {f}_j^{\mathrm{min}}+{k}_{\mathrm{att}}\left({f}_j^{WT}-{f}_j^{\mathrm{min}}\right)\kern1em $$

OR

$$ \left(\mathrm{C}2\right):\kern0.5em {f}_j^{+}+{f}_j^{-}\ge {f}_j^{WT}+{k}_{\mathrm{int}}\left({f}_j^{\mathrm{max}}-{f}_j^{WT}\right)\kern1em $$

Where, constraints (C1) and (C2) are applicable for upregulation and downregulation problems, respectively. Parameters *k*_*att*_ and *k*_*int*_ are gradually varied between 0 and 1 in steps of 0.1 to analyze the effect of reaction upregulation between minimal and wild-type values, and reaction downregulation between the wild-type and maximal values, respectively.

Finally, the objective value obtained from the solution of (Eq. 3) is used as the lower limit for cell growth in the fourth step whereby (Eq. 3) is solved again with the targeted product as the objective function. The corresponding mathematical formulation is as follows:

$$ \min \kern1em {v}_{EX\_ succ} $$

Subject to:

$$ {v}_{biomass}\ge {B}_{j,k} $$

$$ \sum \limits_j{S}_{ij}{v}_j=0 $$

$$ {\alpha}_j\le {v}_j\le {\beta}_j $$

$$ v{}_j={f}_j^{+}-{f}_j^{-} $$

$$ {f}_j^{+}\ge 0\kern1em ;\kern1em {f}_j^{-}\ge 0 $$

$$ {f}_j^{+}\le {I}_j^{+}\cdot M\kern1em ;\kern1em {f}_j^{-}\le {I}_j^{-}\cdot M $$

$$ {I}_j^{+}\in \left\{0,1\right\}\kern1em ;\kern1em {I}_j^{-}\in \left\{0,1\right\} $$

$$ {I}_j^{+}+{I}_j^{-}=1 $$

$$ \left(\mathrm{C}1\right):\kern0.5em {f}_j^{+}+{f}_j^{-}\le {f}_j^{\mathrm{min}}+{k}_{\mathrm{att}}\left({f}_j^{WT}-{f}_j^{\mathrm{min}}\right)\kern1em $$

OR

$$ \left(\mathrm{C}2\right):\kern0.5em {f}_j^{+}+{f}_j^{-}\ge {f}_j^{WT}+{k}_{\mathrm{int}}\left({f}_j^{\mathrm{max}}-{f}_j^{WT}\right)\kern1em $$

where *B*_*j,k*_ is the maximum biomass obtainable while solving problem (Eq. 3) for *j*^th^ reaction at *k*^th^ upregulation/downregulation levels. All the optimization problems were solved using the GAMS IDE software version 22.4 with IBM ILOG CPLEX solver.

#### Transcriptomics-based strain optimization tool (tSOT)

Since optimality assumption based FBA algorithms ignores the regulatory consideration while strain designing, we implemented transcriptomics-based strain optimization tool (tSOT) [35] to identify metabolic engineering targets based on transcriptomic data integrated in the model. A comprehensive time-course transcriptomic profile from the culture of *Y. lipolytica*, during a controlled fed-batch on glucose, was used as transcriptomic data obtained from Gene Expression Omnibus (GEO) database using accession number GSE29046 [40].

#### Cofactor modification analysis (CMA)

Since the ω-oxidation pathway is an oxidative process and requires cofactor optimization to maximize theoretical yield, we used CMA to identify the cofactor specificity engineering target which can increase the yield of DDDA. CMA was implemented as described in our previous work [36]. Mathematically, the bi-level mixed-integer nonlinear programming (MINLP) optimization problem specific to the CMA can be represented as follows:

$$ {\displaystyle \begin{array}{l}\max\ {\varphi}_{product}=0.5\sum \limits_j\left|{S}_{product,j}{v}_j\right|\\ {}\mathrm{s}.\mathrm{t}.\left[\begin{array}{l}\max\ {v}_{biomass}\\ {}\mathrm{s}.\mathrm{t}.\kern1em \sum \limits_j\left({S}_{ij}{v}_j+{S}_{ij}^{cMod}{v}_j^{cMod}\right)=0\forall \mathrm{metabolite}\ i\\ {}\kern2em {v}_{biomass}\ge {v}_{biomass}^{\mathrm{min}}\\ {}\kern2em \left(1-{y}_j^{cMod}\right)\cdot {v}_j^{\mathrm{min}}\le {v}_j\le \left(1-{y}_j^{cMod}\right)\cdot {v}_j^{\mathrm{max}}\\ {}\kern2.25em {y}_j^{cMod}\cdot {v}_j^{\mathrm{min}}\le {v}_j^{cMod}\le {y}_j^{cMod}\cdot {v}_j^{\mathrm{max}}\\ {}\kern2.25em {y}_j^{cMod}=\left\{0,1\right\}\forall \mathrm{reaction}\ j\end{array}\right]\\ {}\sum \limits_j{y}_j^{cMod}\le k\end{array}} $$

Where, ${S}_{ij}^{cMod}$ is the cofactor modified stoichiometric matrix where the coefficients are same as *S*_*ij*_, except the reactions which involve either NAD(H) or NADP(H). These reactions are swapped for cofactors in the ${S}_{ij}^{cMod}$ matrix such that ${S}_{NAD(H),j}={S}_{NAD P(H),j}^{cMod}$ and ${S}_{NAD P(H),j}={S}_{NAD(H),j}^{cMod}$. ${v}_j^{cMod}$ is the flux through the cofactor modified reaction and ${v}_{biomass}^{\mathrm{min}}$ is the minimum amount of biomass that needs to be produced. The binary variable ${y}_j^{cMod}$ ensures that the cofactor associated reactions are allowed to carry flux either with its original or swapped cofactor but not both. The number of cofactor switches allowed in a particular simulation is controlled by the number, *k* and which is fixed at 1 for all simulations in this work. The bi-level MINLP problem was reformulated as a single-level MINLP problem using the primal dual transformation as implemented earlier [58]. The MILP optimization problem was solved using the GAMS IDE software version 22.4 with IBM ILOG CPLEX solver.

### Flux-sum

In the constraints-based flux analysis, there is no accumulation of intermediate metabolites due to the steady-state condition. However, the turnover rate, which is also equivalent to the total consumption or production rate, of the intermediates can be nonzero, which is defined as their flux-sum [59]. Since the overall consumption and generation rates are equal under the steady-state assumption, the flux-sum of metabolite *i* can be formulated as Φ_*i*_ = 0.5 ∑ |*S*_*ij*_*v*_*j*_|. Each term in this summation series gives us the absolute rate of consumption/generation of metabolite *i* due to reaction *j* and thus by halving the sum of these terms, we can obtain the overall turnover rate for metabolite *i*.

## Additional files

Additional file 1: Changed, added and deleted reactions in *i*YLI647 model in comparison with *i*MK735 scaffold model. (XLSX 22 kb)

Additional file 2: SMBL file of *i*YLI647. (XML 2312 kb)

Additional file 3: Biomass composition of *Y. lipolytica* in C- and N- limited conditions and GAM and NGAM calculations. (DOCX 52 kb)
