# Supplementary material for: Genome-scale model-driven strain design for dicarboxylic acid production in Yarrowia lipolytica
Source: BMC Syst Biol. 2018 Mar 19;12(Suppl 2):12. doi: 10.1186/s12918-018-0542-5 (PMC5861505; doi:10.1186/s12918-018-0542-5)
Supplement: Supplementary file 3 — Biomass composition of Y. lipolytica in C- and N- limited conditions and GAM and NGAM calculations. (DOCX 52 kb) [file 12918_2018_542_MOESM3_ESM.docx]

**Biomass composition in C-limited condition**

| Table S1. Overall macromolecular composition of *Y. lipolytica* (Zhang et al., 2016) | | |
| --- | --- | --- |
| Component | Cellular content% (w/w) | GAM (mmol ATP/gDCW) |
| Protein | 40.3 | 16.7245 |
| Carbohydrates | 33.5 | 4.288 |
| RNA | 6.8 | 1.768 |
| DNA | 1.2 | 0.312 |
| Lipid | 8.7 |  |
| Others | 9.5 |  |
| Total | 100 | 23.09 |

| Table S2. Amino acid composition of *Y. lipolytica* (Zhang et al., 2016) | | |  |
| --- | --- | --- | --- |
| Amino acid | Composition (% of total protein) | MW (g/mol) | Content (mmol/gDCW) |
| Alanine | 11.5 | 71 | 0.097846697 |
| Arginine | 5 | 157 | 0.019238758 |
| Asparagine | 5.6 | 114 | 0.02967494 |
| Aspartate | 5.6 | 114 | 0.02967494 |
| Cysteine | 0.1 | 103 | 0.000586502 |
| Glutamine | 8.1 | 128 | 0.038228013 |
| Glutamate | 8.1 | 128 | 0.038228013 |
| Glycine | 8.7 | 57 | 0.092204279 |
| Histidine | 1.9 | 137 | 0.008377988 |
| Isoleucine | 2.7 | 113 | 0.014434176 |
| Leucine | 5.8 | 113 | 0.031006749 |
| Lysine | 9.2 | 129 | 0.043082887 |
| Methionine | 1.5 | 131 | 0.006917141 |
| Phenylalanine | 3.1 | 147 | 0.012739461 |
| Proline | 5.4 | 97 | 0.033630142 |
| Serine | 6.8 | 87 | 0.047216777 |
| Threonine | 5.7 | 101 | 0.034092603 |
| Tryptophan | 0.1 | 186 | 0.000324783 |
| Tyrosine | 1.7 | 163 | 0.006300398 |
| Valine | 4.6 | 99 | 0.028069154 |

| Table S3. RNA composition of 1 gram of *Y. lipolytica* cell (Feist et al., 2007) | | | |
| --- | --- | --- | --- |
| Metabolite | mRNA (%) | MW (g/mol) | Content (mmol/g DCW) |
| cmp | 0.245 | 323 | 0.007731684 |
| gmp | 0.245 | 363 | 0.006879708 |
| ump | 0.255 | s324 | 0.008022426 |
| amp | 0.255 | 347 | 0.00749068 |
|  |  |  |  |

| Table S4. DNA composition of 1 gram of *Y. lipolytica* cell (GC content of *Y. lipolytica* is 49%) (Zhang et al., 2016) | | | |
| --- | --- | --- | --- |
| Metabolite | Composition (%) | MW (g/mol) | Content (mmol/g DCW) |
| damp | 0.255 | 331 | 0.001385782 |
| dcmp | 0.245 | 307 | 0.001435524 |
| dgmp | 0.245 | 347 | 0.001270046 |
| dtmp | 0.255 | 322 | 0.001424516 |

| Table S5. Carbohydrate composition of *Y. lipolytica* (Xue et al., 2013) | | | |
| --- | --- | --- | --- |
| Carbohydrate | Content in dry cell(mg/g) | MW (g/mol) | Content (mmol/gDCW) |
| chitin(monomer) | 52.6 | 203 | 0.259113 |
| Trehalose | 0.7 | 342 | 0.002047 |
| Mannan | 11.4 | 162 | 0.07037 |
| glucan | 45.6 | 162 | 0.281481 |

| Table 6. Fatty acid composition of *Y. lipolytica* (Zhang et al., 2016) | | | |
| --- | --- | --- | --- |
|  |  |  |  |
| Fatty Acid | Content %(w/w) | MW (g/mol) | mol/g FA |
| C16:0 | 9.789607019 | 255.4 | 0.000383305 |
| C16:1 | 5.929269577 | 253.4 | 0.000233989 |
| C18:0 | 2.366317961 | 283.5 | 8.3468E-05 |
| C18:1 | 48.66082548 | 281.5 | 0.001728626 |
| C18:2 | 33.25397996 | 279.5 | 0.001189767 |
| Avg MW of FA |  |  | 276.3076452 |

| Table 7. Detailed lipid composition of *Y. lipolytica* (Kerkhoven et al., 2016) | | | |
| --- | --- | --- | --- |
| Lipid | Content (mg/gDCW) | MW (g/mol) | Content (mmol/gDCW) |
| TAG(Triacylglycerol) | 9.880239521 | 912.758 | 0.023403363 |
| E(ergosterol) | 6.467065868 | 396.6484 | 0.035250723 |
| SE(steryl esters : zymosterol) | 0.538922156 | 384.6377 | 0.003029289 |

| Table 8. Phospholipid content of *Y. lipolytica* (Kerkhoven et al., 2016) | |  |
| --- | --- | --- |
| Phospholipids | Composition (mg/gDCW) | Content (mmol/gDCW) |
| PA (Phosphatidate) | 1.796407186 | 0.005774757 |
| PINS (phosphatidyl-1D-myo-inositol) | 2.335329341 | 0.007507185 |
| PS | 1.077844311 | 0.003464854 |
| PE | 7.365269461 | 0.023676505 |
| PC | 10.77844311 | 0.034648544 |

Final biomass equation in C-limited condition

0.281481 13BDglcn[c] + 0.0978466971830986 ala_L[c] + 0.0482 amp[c] + 0.0192387579617834 arg_L[c] + 0.0296749403508772 asn_L[c] + 0.0296749403508772 asp_L[c] + 23.09 atp[c] + 0.0368 cmp[c] + 0.000586501941747573 cys_L[c] + 0.00138578247734139 damp[c] + 0.00143552442996743 dcmp[c] + 0.00127004610951009 dgmp[c] + 0.00142451552795031 dtmp[c] + 0.035250723 ergst[c] + 0.03822801328125 gln_L[c] + 0.03822801328125 glu_L[c] + 0.0922042789473684 gly[c] + 0.259113 chitin[c] + 0.0593 gmp[c] + 23.09 h2o[c] + 0.00837798759124088 his_L[c] + 0.0144341761061947 ile_L[c] + 0.0310067486725664 leu_L[c] + 0.0430828868217054 lys_L[c] + 0.07037 mannan[c] + 0.00691714122137404 met_L[c] + 0.00005774757 pa_SC[c] + 0.00034648544 pc_SC[c] + 0.00023676505 pe_SC[c] + 0.0127394605442177 phe_L[c] + 0.0336301422680412 pro_L[c] + 0.00003464854 ps_SC[c] + 0.00007507185 ptd1ino_SC[c] + 0.0472167770114943 ser_L[c] + 0.02 so4[c] + 0.034092602970297 thr_L[c] + 0.002047 tre[c] + 0.00023403363 triglyc_SC[c] + 0.000324783333333333 trp_L[c] + 0.0063003981595092 tyr_L[c] + 0.0397 ump[c] + 0.0280691535353535 val_L[c] + 0.003029289 zymst[c] -> 23.09 adp[c] + 23.09 pi[c] + 23.09 h[c]

**Biomass composition in N-limited condition**

| Table 1. Overall Macromolecular composition of *Y. lipolytica* (Zhang et al., 2016) | | |
| --- | --- | --- |
| Component | Cellular content% (w/w) | GAM (mmol ATP/gDCW) |
| Protein | 35.7 | 14.8155 |
| Carbohydrates | 30.6 | 3.9168 |
| RNA | 4.8 | 1.248 |
| DNA | 1.3 | 0.338 |
| Lipid | 14.3 |  |
| Others | 13.3 |  |
| Total | 100 | 20.31 |

| Table 2. Amino acid composition of *Y. lipolytica* (Zhang et al., 2016) | | |  |
| --- | --- | --- | --- |
| Amino acid | Composition (% of total protein) | MW (g/mol) | Content (mmol/gDCW) |
| Alanine | 12.6 | 71 | 0.094969039 |
| Arginine | 5.3 | 157 | 0.018065337 |
| Asparagine | 5.6 | 114 | 0.026287726 |
| Aspartate | 5.6 | 114 | 0.026287726 |
| Cysteine | 0.1 | 103 | 0.000519556 |
| Glutamine | 7.7 | 128 | 0.032192196 |
| Glutamate | 7.7 | 128 | 0.032192196 |
| Glycine | 8.8 | 57 | 0.082618568 |
| Histidine | 1.8 | 137 | 0.007031076 |
| Isoleucine | 2.9 | 113 | 0.013733758 |
| Leucine | 6.1 | 113 | 0.02888825 |
| Lysine | 7.6 | 129 | 0.031527805 |
| Methionine | 0.7 | 131 | 0.002859543 |
| Phenylalanine | 3.3 | 147 | 0.012013414 |
| Proline | 5.2 | 97 | 0.028688078 |
| Serine | 7.6 | 87 | 0.046748124 |
| Threonine | 6 | 101 | 0.031790673 |
| Tryptophan | 0.1 | 186 | 0.000287711 |
| Tyrosine | 1.9 | 163 | 0.006237863 |
| Valine | 4.8 | 99 | 0.025946327 |

| Table 3. RNA Composition of 1 Gram of *Y. lipolytica* Cell (Feist et al., 2007) | | | |
| --- | --- | --- | --- |
| Metabolite | mRNA | MW (g/mol) | Content (mmol/g DCW) |
| cmp | 0.245 | 323 | 0.001478116 |
| gmp | 0.245 | 363 | 0.001315238 |
| ump | 0.255 | 324 | 0.001533699 |
| amp | 0.255 | 347 | 0.001432042 |
|  |  |  |  |

| Table 4. DNA Composition of 1 Gram of *Y. lipolytica* Cell (GC content of *Y. lipolytica* is 49%) (Zhang et al., 2016) | | | |
| --- | --- | --- | --- |
| Metabolite | Composition (molar fraction) | MW (g/mol) | Content (mmol/g DCW) |
| damp | 0.255 | 331 | 0.016513908 |
| dcmp | 0.245 | 307 | 0.017106666 |
| dgmp | 0.245 | 347 | 0.015134716 |
| dtmp | 0.255 | 322 | 0.016975477 |

| Table 5. Carbohydrate composition of *Y. lipolytica* (Xue et al., 2013) | | | | | | |
| --- | --- | --- | --- | --- | --- | --- |
| Carbohydrate | Content in dry cell(mg/g) | | MW (g/mol) | | Content (mmol/gDCW) | |
| chitin(monomer) | 52.6 | | 203 | | 0.236682322 | |
| Trehalose | 0.7 | | 342 | | 0.001869797 | |
| Mannan | 11.4 | | 162 | | 0.064278269 | |
| glucan | 45.6 | | 162 | | 0.257113988 | |
| Table 6. Fatty acid composition of *Y. lipolytica* (Zhang et al., 2016) | | | | | | |
|  | |  | |  | |  |
| Fatty Acid | | Content %(w/w) | | MW (g/mol) | | mol/g FA |
| C16:0 | | 10.32515551 | | 255.4 | | 0.000404274 |
| C16:1 | | 8.354765148 | | 253.4 | | 0.000329707 |
| C18:0 | | 2.744364422 | | 283.5 | | 9.6803E-05 |
| C18:1 | | 56.58024994 | | 281.5 | | 0.002009956 |
| C18:2 | | 21.99546498 | | 279.5 | | 0.000786958 |
| Avg. MW of FA | |  | |  | | 275.657005 |

| Table 7. Detailed lipid composition of *Y. lipolytica* (Kerkhoven et al., 2016) | | | | | |
| --- | --- | --- | --- | --- | --- |
|  | | | | | |
| Lipid | Content (mg/gDCW) | | MW (g/mol) | | Content (mmol/gDCW) |
| TAG(Triacylglycerol) | 47.25111441 | | 912.758 | | 0.038910587 |
| E(ergosterol) | 32.00594354 | | 396.6484 | | 0.060650767 |
| SE(steryl esters : zymosterol) | 1.783060921 | | 384.6377 | | 0.003484382 |
| Table 8. Phospholipid content of *Y. lipolytica* (Kerkhoven et al., 2016) | | | |  | |
| Phospholipids | | Composition (mg/gDCW) | | Content (mmol/gDCW) | |
| PA (Phosphatidate) | | 7.578008915 | | 0.008468939 | |
| PINS (phosphatidyl-1D-myo-inositol) | | 11.2332838 | | 0.012553956 | |
| PS | | 9.806835067 | | 0.010959803 | |
| PE | | 28.88558692 | | 0.057788053 | |
| PC | | 51.70876672 | | 0.057788053 | |

Final biomass equation in N-limited condition

0.257113988 13BDglcn[c] + 0.09496 ala_L[c] +0.001432042 amp[c] + 0.018065337 arg_L[c] + 0.026287726 asn_L[c] + 0.026287726 asp_L[c] + 20.31 atp[c] + 0.001478116 cmp[c] + 0.000519556 cys_L[c] + 0.016513908 damp[c] + 0.017106666 dcmp[c] + 0.015134716 dgmp[c] + 0.016975477 dtmp[c] + 0.060650767 ergst[c] + 0.032192196 gln_L[c] + 0.032192196 glu_L[c] +0.082618568 gly[c] + 0.236682322 chitin[c] + 0.001315238 gmp[c] + 20.31 h2o[c] + 0.007031076 his_L[c] + 0.013733758 ile_L[c] + 0.02888825 leu_L[c] + 0.031527805 lys_L[c] + 0.064278269 mannan[c] + 0.002859543 met_L[c] + 0.00008468939 pa_SC[c] + 0.00057788053 pc_SC[c] + 0.00057788053 pe_SC[c] + 0.012013414 phe_L[c] + 0.028688078 pro_L[c] + 0.00010959803 ps_SC[c] + 0.00012553956 ptd1ino_SC[c] + 0.046748124 ser_L[c] + 0.02 so4[c] + 0.031790673 thr_L[c] + 0.001869797 tre[c] + 0.00038910587 triglyc_SC[c] + 0.000287711 trp_L[c] + 0.006237863 tyr_L[c] + 0.001533699 ump[c] + 0.025946327 val_L[c] + 0.003484382 zymst[c] -> 20.31 adp[c] + 20.31 pi[c] + 20.31 h[c]

**Non-growth associated ATP maintenance (NGAM) requirement**

The NGAM is the quantum of ATP required for the maintenance of cell machinery even when not actively dividing. It is predominantly used for maintaining the membrane potential. In this study, we determined the NGAM requirement from the chemostat experiment using a conventional method of finding the y-intercept of the plot of glycerol uptake rate against dilution rate (Pirt, 1982).

By maximizing ATP turnover under the glycerol uptake constraint of 1 mmol/gDCW-hr, the ATP yield is evaluated as Y_ATP_, max = 6 mol ATP/ mol glycerol. Using this value and the y-intercept (0.8387 mmol glycerol/gDCW-hr), we can calculate the NGAM requirement to be about 5.03 mmol ATP/gDCW-hr.

**References**

Feist AM, Henry CS, Reed JL, Krummenacker M, Joyce AR, Karp PD, Broadbelt LJ, Hatzimanikatis V, Palsson BØ. 2007. A genome-scale metabolic reconstruction for Escherichia coli K-12 MG1655 that accounts for 1260 ORFs and thermodynamic information. *Mol. Syst. Biol.* **3**:121.

Kerkhoven EJ, Pomraning KR, Baker SE, Nielsen J. 2016. Regulation of amino-acid metabolism controls flux to lipid accumulation in Yarrowia lipolytica. *npj Syst. Biol. Appl.* **in press**:1–7.

Pirt SJ. 1982. Maintenance energy: a general model for energy-limited and energy-sufficient growth. *Arch. Microbiol.* **133**:300–302.

Xue Z, Sharpe PL, Hong S-P, Yadav NS, Xie D, Short DR, Damude HG, Rupert RA, Seip JE, Wang J, Pollak DW, Bostick MW, Bosak MD, Macool DJ, Hollerbach DH, Zhang H, Arcilla DM, Bledsoe SA, Croker K, McCord EF, Tyreus BD, Jackson EN, Zhu Q. 2013. Production of omega-3 eicosapentaenoic acid by metabolic engineering of Yarrowia lipolytica. *Nat. Biotechnol.* **31**:734–740.

Zhang H, Wu C, Wu Q, Dai J, Song Y. 2016. Metabolic flux analysis of lipid biosynthesis in the yeast Yarrowia lipolytica using 13C-labled glucose and gas chromatography-mass spectrometry. *PLoS One* **11**.
